# Beckwith–Wiedemann syndrome with juvenile fibrous nodules and lobular breast tumors: a case report and review of the literature

**DOI:** 10.1186/s40792-024-01865-2

**Published:** 2024-03-22

**Authors:** Yo Sato, Yusuke Watanabe, Takafumi Morisaki, Saori Hayashi, Yoshiki Otsubo, Yurina Ochiai, Kimihisa Mizoguchi, Yuka Takao, Mai Yamada, Yusuke Mizuuchi, Masafumi Nakamura, Makoto Kubo

**Affiliations:** 1 Departments of Surgery and Oncology, Graduate School of Medical Sciences, Kyushu University Hospital, 3-1-1, Maidashi, Higashi-Ku, Fukuoka, 812-8582 Japan; 2 Department of Breast Surgical Oncology, Kyushu University Hospital, 3-1-1, Maidashi, Higashi-Ku, Fukuoka, 812-8582 Japan; 3 Department of Clinical Genetics and Medicine, Kyushu University Hospital, Fukuoka, Japan

**Keywords:** Beckwith–Wiedemann syndrome, Hemihypertrophy, Fibrous nodules and lobular breast tumor, Insulin-like growth factor-2

## Abstract

**Background:**

Beckwith–Wiedemann syndrome (BWS) is a genomic imprinting disorder caused by diverse genetic and/or epigenetic disorders of chromosome 11p15.5. BWS presents with a variety of clinical features, including overgrowth and an increased risk of embryonal tumors. Notably however, reports of patients with BWS and breast tumors are rare, and the association between these conditions is still unclear. Insulin-like growth factor-2 (IGF2) expression is known to be associated with the development of various cancers, including breast cancer, and patients with BWS with specific subtypes of molecular defects are known to show characteristic clinical features and IGF2 overexpression.

**Case presentation:**

A 17-year-old girl who had been diagnosed with BWS based on an umbilical hernia, hyperinsulinemia, and left hemihypertrophy at birth, visited our department with a gradually swelling left breast. Her left breast was markedly larger than her right breast on visual examination. Imaging examinations showed two tumors measuring about 10 cm each in the left breast, and she was diagnosed with juvenile fibroadenoma following core needle biopsy. The two breast tumors were removed surgically and the patient remained alive with no recurrence. The final diagnosis was juvenile fibroadenoma without malignant findings. Immunohistochemical staining using IGF2 antibody revealed overexpression of IGF2 in the cytoplasm of ductal epithelial cells. Because of her clinical features and IGF2 overexpression, molecular defects of 11p15.5 including a possible genetic background of paternal uniparental disomy of chromosome 11 or hypermethylation of imprinting center 1 was suspected.

**Conclusions:**

In this case, overexpression of IGF2 suggested a possible relationship between BWS and breast tumors. Moreover, the characteristic clinical features and IGF2 staining predicted the subtype of 11p15.5 molecular defects in this patient.

## Background

Beckwith–Wiedemann syndrome (BWS) is a genomic imprinting disorder that presents with a variety of clinical features, including umbilical hernia, macroglossia, gigantism, neonatal hypoglycemia, lateral hypertrophy, ear anomalies, and an increased risk of embryonal tumors. BWS can be caused by various genetic and/ or epigenetic alterations that usually affect the regulation of genes imprinted on chromosome 11p15.5, resulting in a heterogeneous clinical spectrum [1]. About 80% of patients with BWS have a known molecular defect in the 11p15 region as a result of aberrant DNA methylation, mosaic paternal uniparental disomy (pUPD), or mutations in the *CDKN1C* gene, with each of these defects having characteristic clinical features [1]. Several mechanisms may lead to increased expression of insulin-like growth factor-2 (IGF2) in patients with BWS, and IGF2 has been associated with tumorigenesis in various breast tumors [5]; however, few reports have described fibroadenomas in patients with BWS [2–7].

Herein, we report the case of a patient with BWS who had giant juvenile fibroadenomas, which were removed surgically, associated with overexpression of IGF2.

## Case presentation

A 17-year-old girl diagnosed with BWS was referred to our department because of rapidly growing masses in her left breast. Regarding her medical history, she had been diagnosed with BWS at birth. High-resolution chromosome banding indicated no structural abnormalities of chromosome 11; however, she had an umbilical hernia, hyperinsulinemia, and left hemihypertrophy, leading to a final clinical diagnosis of BWS. She had no family history of hereditary disease, including BWS.

**Fig. 1:**
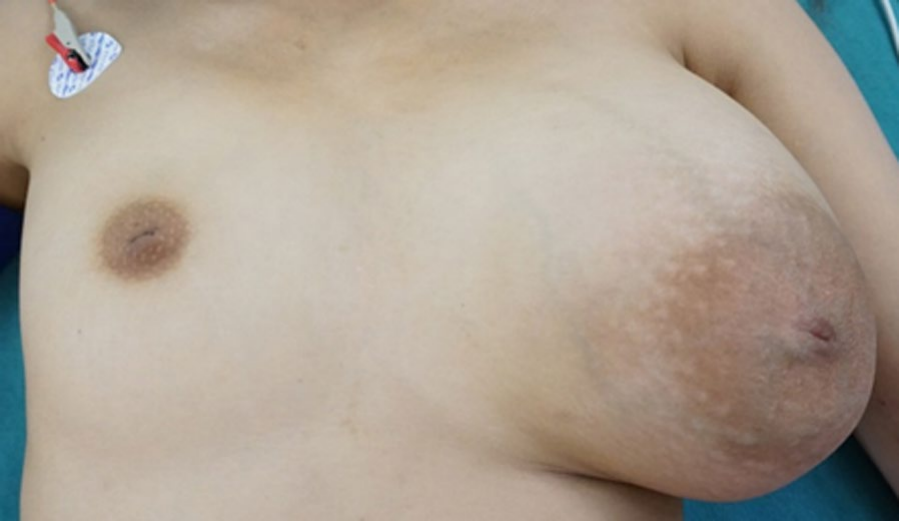
Physical examination findings. The patient’s left breast was markedly larger than her right breast, with two palpable masses measuring approximately 10 cm each

**Fig. 2:**
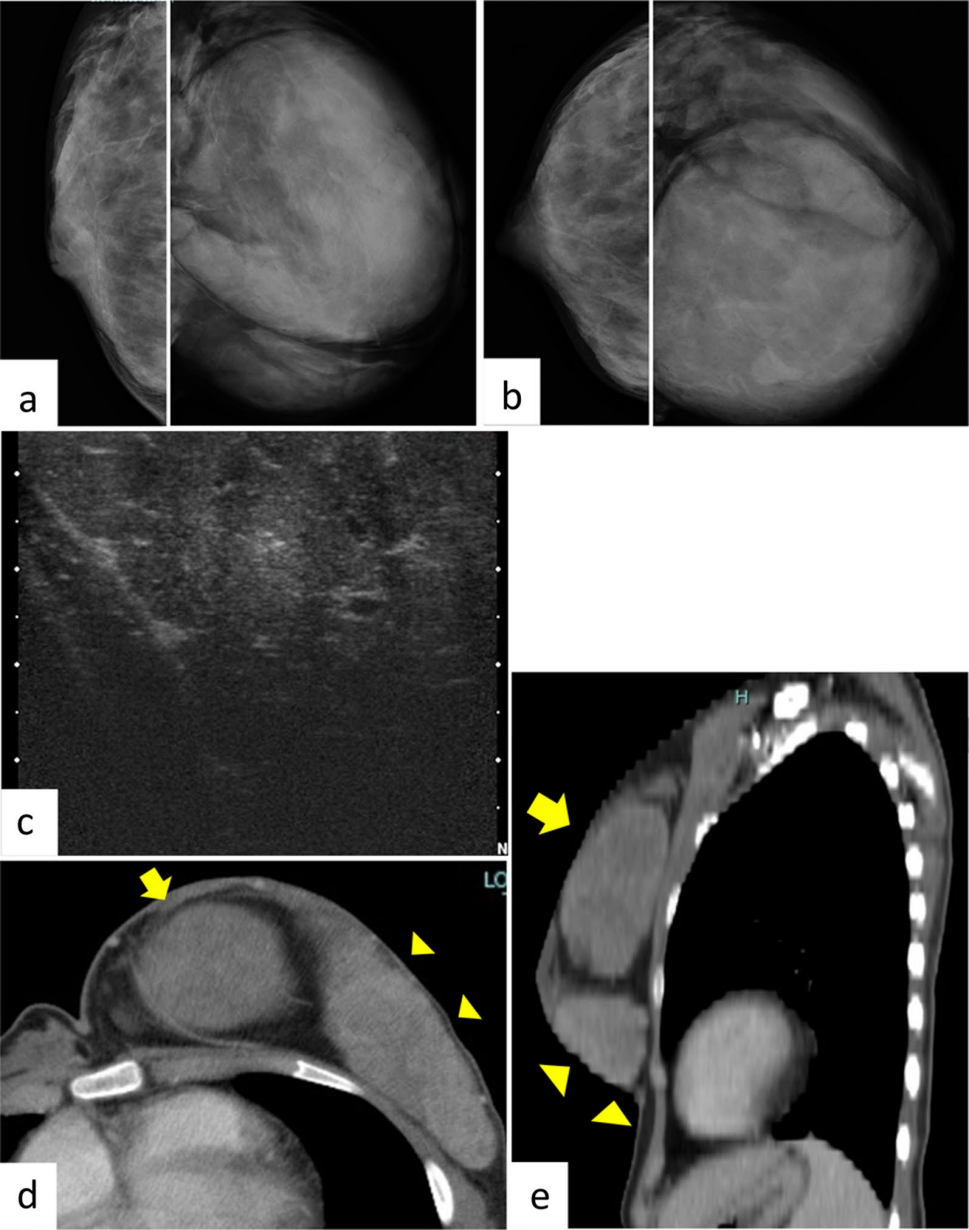
Imaging findings.** a**, **b** Mammography revealed a well-demarcated huge mass occupying the entire left breast (Breast Imaging Reporting and Data System category 3). **a** Medio-lateral oblique view; **b** cranio-caudal view. **c** Ultrasound showed two giant, well-demarcated, isoechoic lesions in the left breast, with homogeneous tumor parenchyma. **d**, **e** Contrast-enhanced computed tomography showed two well-demarcated masses in the left breast. Arrow indicates upper-inner lesion; arrowheads indicate lateral lesions. **d** Axial view; **e** sagittal view

**Fig. 3:**
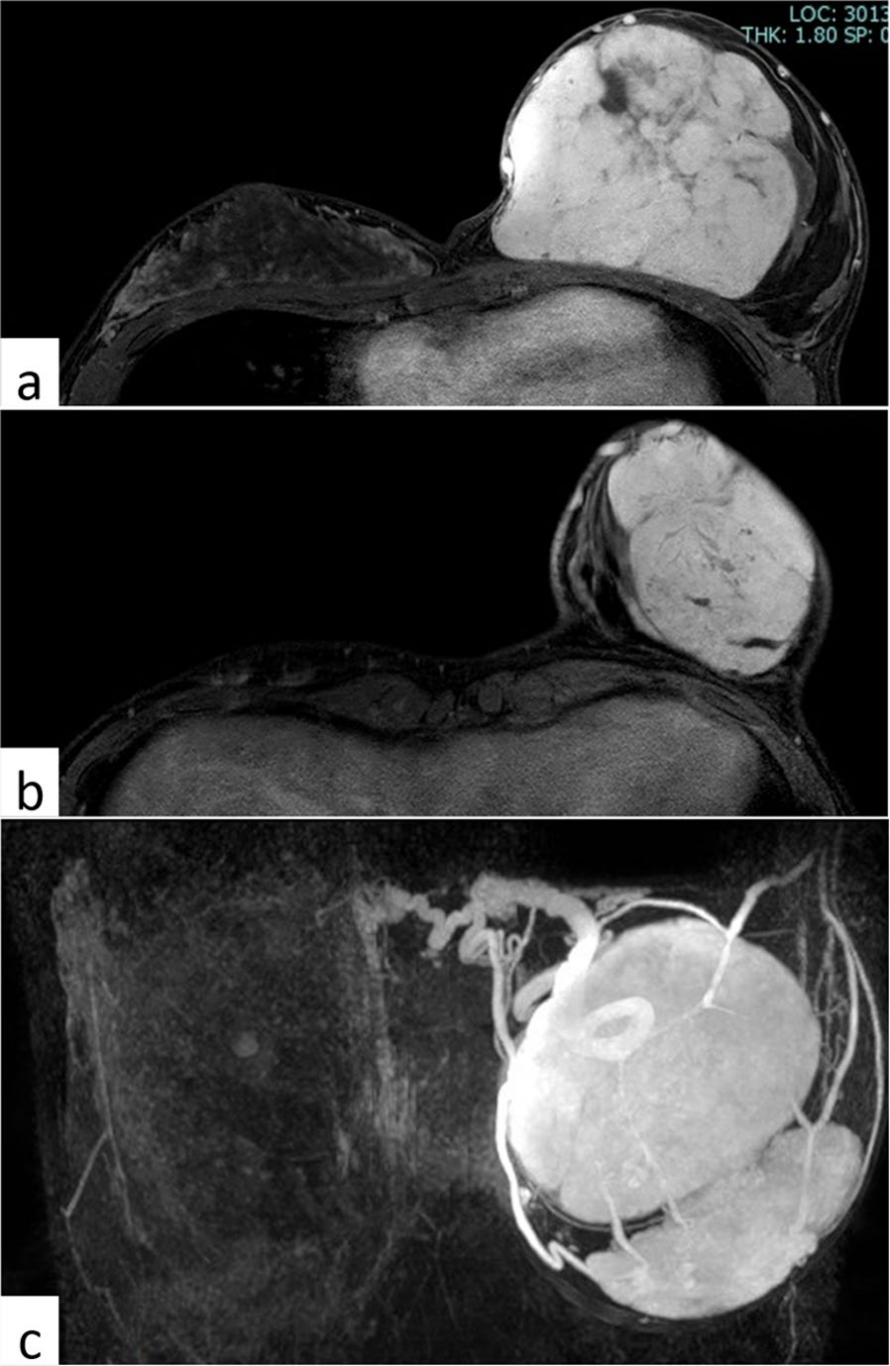
Contrast-enhanced magnetic resonance imaging. **a** Well-defined 10 cm mass occupying the upper part of the left breast and **b** 7.7 cm mass in the lower-outer part of the left breast. **c** The two masses were fed by the lateral thoracic and internal thoracic arteries and the drainage veins were remarkably dilated

**Fig. 4:**
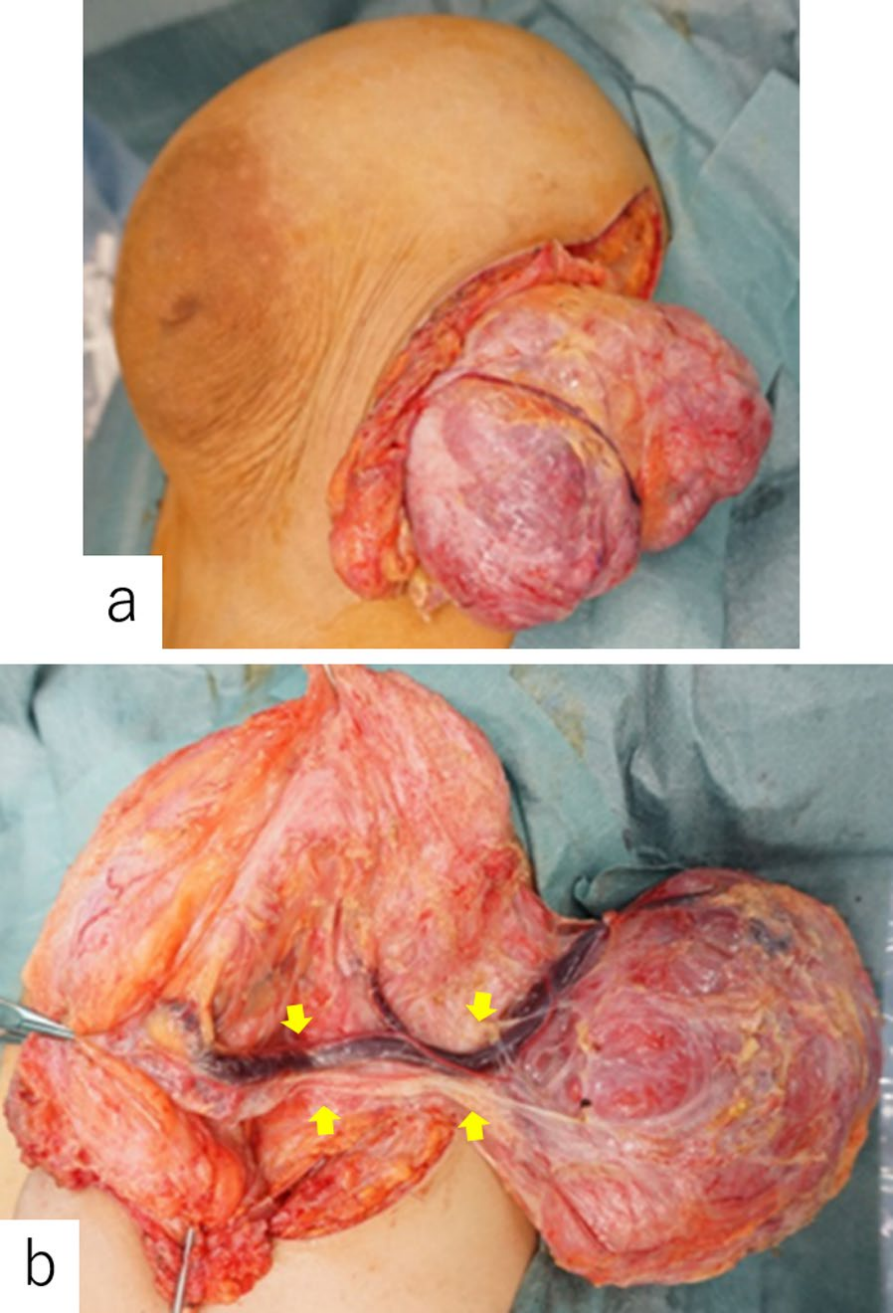
Intraoperative findings. **a** A skin incision was made on the lateral side of the left breast and two masses were enucleated. **b** The masses were fed by the internal thoracic artery and dilated internal thoracic vessels (arrows)

Physical examination at initial consultation revealed left-sided hemihypertrophy. Her left breast was markedly larger than her right breast, with two palpable masses, each measuring approximately 10 cm (Fig. 1). Laboratory data, including tumor markers, were within normal limits. A mammogram showed two well-demarcated masses occupying the entire left breast (Fig. 2a, b), ultrasound revealed two giant well-demarcated isoechoic lesions in the upper-inner and outer areas of the left breast, respectively (Fig. 2c), and contrast-enhanced computed tomography showed two heterogeneous enhanced masses of about 10 cm in the left breast (Fig. 2d, e). No enlarged axial or supraclavicular lymph nodes were detected. Contrast magnetic resonance imaging showed two well-defined masses measuring about 10 and 8 cm, respectively, in the left breast, with progressive enhancement in dynamic imaging (Fig. 3a, b). The contrast pattern suggested fibroadenoma or phyllodes tumor. The two masses were fed by the lateral thoracic and internal thoracic arteries, and the drainage veins surrounding the breast tumors were markedly dilated (Fig. 3c). Examination of a core needle biopsy specimen showed benign breast tissue with a proliferation of branching mammary ducts and spindle-shaped stromal cells, indicating a fibroadenoma. The lesions were accordingly diagnosed as fibroadenoma with rapid growth and the patient underwent surgical resection. A skin incision was made on the lateral side of the left breast under general anesthesia. The feeding vessels were transected carefully and the two masses were enucleated (Fig. 4a, b). The operative duration was 145 min and the blood loss was 6 g.

**Fig. 5:**
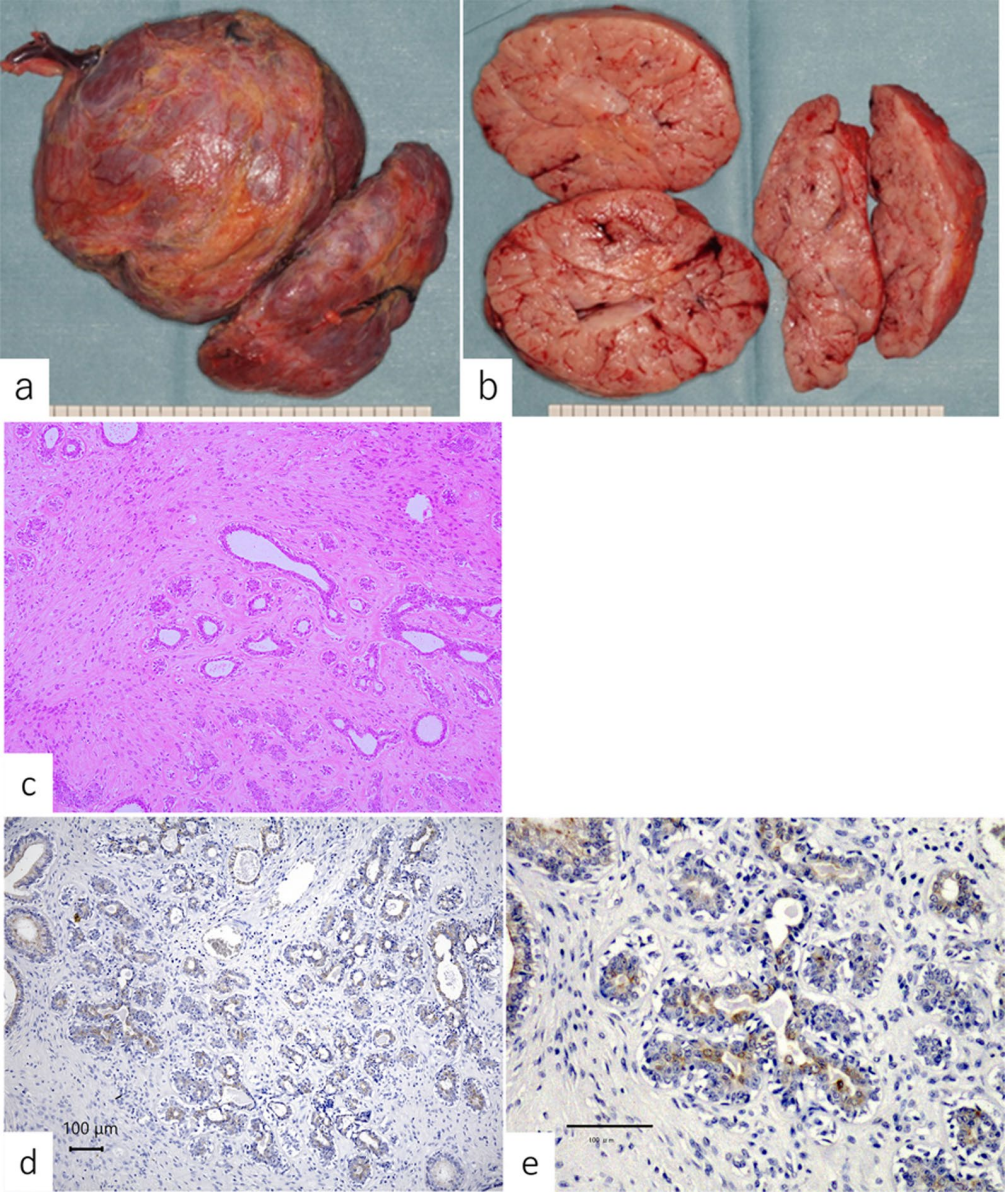
Macroscopic and microscopic findings of resected specimens. **a** Macroscopically, the resected tumors were smooth and well-circumscribed masses measuring about 11 cm in the upper-inner quadrant and 14 cm in the outer breast, respectively. **b** Lobulated lesions with slit-like spaces were observed in the cut surface. **c** Microscopically, sections revealed a proliferation of branching mammary ducts and hyperplastic lobules and spindle-shaped stromal cells, accompanied by hyalinized or fibro-myxoid stroma (hematoxylin–eosin staining, × 40). The features indicated juvenile fibroadenomas. There was no evidence of malignancy. **d**, **e** Immunohistochemical staining with insulin-like growth factor-2 (IGF2) antibody showed overexpression of IGF2 in the cytoplasm of ductal epithelial cells of the fibroadenoma. (**d**  × 40, **e**  × 100)

The macroscopic findings of the resected specimens revealed smooth, well-circumscribed masses, and lobulated lesions with slit-like spaces were observed in the cut surface (Fig. 5a, b). Microscopically, a proliferation of branching mammary ducts, hyperplastic lobules, and spindle-shaped stromal cells accompanied by hyalinized or fibro-myxoid stroma were observed, indicating fibroadenoma (Fig. 5c). The lesions were accordingly diagnosed as juvenile fibroadenomas.　Immunohistochemical staining using IGF2 antibody revealed overexpression of IGF2 in the cytoplasm of the ductal epithelial cells of the fibroadenoma (Fig. 5d, e).

Postoperatively, the patient recovered uneventfully and was discharged on postoperative day 2. She had experienced no recurrence of the breast tumors at the time of this report, 9 years after the operation.

## Discussion

BWS is a heterogeneous overgrowth syndrome caused by diverse genetic and/or epigenetic disorders, usually affecting the regulation of genes imprinted on chromosome 11p15. BWS was initially reported by Beckwith in 1963 and Hans-Rudolf Wiedemann in 1964, and the syndrome was subsequently named after these two investigators. BWS is equally represented in males and females, with a reported incidence of approximately 1/10,000–13,700 [1]. Among patients with BWS, 85% have no relevant family history and only a few chromosomal abnormalities of 11p15 have been reported [8]. Most patients have good physical and developmental prognoses, but severe complications including prematurity, giant tongue, or cardiomyopathy can lead to fatal outcomes [9]. The most characteristic clinical features of BWS are exomphalos, macroglossia, and gigantism; however, only 56% of patients develop this triad [10], and other clinical features include ear anomalies, neonatal hypoglycemia, unilateral hypertrophy, organomegaly, cardiac anomalies, and musculoskeletal abnormalities. Hemihypertrophy is known to be associated with tumor development (nephroblastoma, adrenal neoplasm, and liver neoplasm), usually on the ipsilateral side [1].

BWS is diagnosed clinically by the presence of at least three major features, or two major and one minor finding [11], while molecular testing may play an important diagnostic role in children with clinical features of BWS who do not meet the diagnostic criteria. BWS is caused by diverse genetic and/or epigenetic disorders that usually affect the regulation of genes imprinted on chromosome 11p15.5. The most common cause is loss of methylation at independent IC2, which occurs in 50%–60% of all cases. Other causes include pUPD of chromosome 11 (pUPD11) in 20%, gain of methylation at imprinting center (IC1) in about 5% of cases, and mutations in the *CDKN1C* gene in about 5% [8, 12]. BWS is associated with an increased risk of embryonal tumors, such as Wilms tumor and hepatoblastoma, as well as other benign and malignant neoplasms. Hypermethylation of IC1 and pUPD11 result in upregulation of the biallelic expression of IGF2, which has been associated with tumorigenesis in various subtypes of breast tumors [13].

The PubMed database includes six case reports of benign phyllodes tumor and fibroadenoma in patients with BWS (Table 1) [2–7]. All patients were female and five patients had hemihypertrophy, of whom four had lesions located at the ipsilateral side of hemihypertrophy. Two patients experienced recurrence [2, 7]. Of these six cases, chromosomal disorders were only reported in two cases [4, 5]. Takama et al. reported pUPD of chromosome 11p15.5 detected by microsatellite marker analysis, with the possibility to increase IGF2 expression, and which has reportedly been associated with fibroadenoma [5]. Cappuccio et al. reported the results of DNA methylation by combined bisulfite restriction analyses [4]. Although methylation levels at IC1 and IC2 were normal, a single nucleotide polymorphism array revealed a de novo 7p22.1 loss in both blood and breast tumor tissue involving the mismatch repair gene *PMS2*; however, they concluded that the relationship between this molecular defect and the risk of breast tumorigenesis was unclear.

**Table 1:** Case reports of patient with Beckwith–Wiedemann syndrome accompanied with fibroadenoma

Author	Year	Country	Sex	Age	Hemihypertrophy	Location of the lesion*	Tumor size (cm)	Chromosomal disorder
Raine [2]	1979	Australia	F	7m	Presence	Contralateral side	5	NA
Poh [3]	2010	America	F	12yo	Presence	Ipsilateral side	12	NA
Cappuccio [4]	2013	Italy	F	13yo	Presence	Ipsilateral side	6.7 and 5.8	7p22.1 loss
Takama [5]	2014	Japan	F	16yo	Absent	–	NA	pUPD of 11p15.5
Szymanska [6]	2017	Poland	F	11yo	Presence	Ipsilateral side	NA	NA
Oktay [7]	2021	Turkey	F	13yo	Presence	Ipsilateral side	7	NA

*F* female; *yo* years old; *m* month; *NA* not available; *pUPD* paternal uniparental disomy

^*^This indicates a relationship between hemihypertrophy and location of the lesion

In the current case, immunohistochemical staining of IGF2 in surgical breast tissue revealed overexpression of IGF2 in the cytoplasm of ductal epithelial cells. IGF2 expression is reportedly more upregulated in breast tumors including fibroadenoma than in non-neoplastic mammary grand tissue [14]. To the best of our knowledge, this is the first report to indicate a relationship between IGF2 overexpression and benign breast tumors in a patient with BWS. Francisco et al. showed that the clinical features of BWS depended on its molecular defects [1]; patients with pUPD11 often present with hemihypertrophy and tumor development, while patients with hypermethylation of IC1 often present with umbilical hernia and macrosomia. The present case demonstrated hemihypertrophy and umbilical hernia, suggesting possible pUPD11 or hypermethylation of IC1. Both of these molecular defects result in overexpression of IGF2, which may be associated with tumorigenesis in various subtypes of breast cancer and may cause juvenile fibrous and lobular breast tumors, as in the current case. Nevertheless, the association between BWS and the risk of breast lesions and the molecular defects responsible for the occurrence of these breast tumors is still unknown.

## Conclusions

The limited number of reports of patients with BWS and juvenile fibroadenoma means that the relationship between breast tumors and BWS is still unclear. Overexpression of IGF2, which may be associated with the occurrence of breast tumors, was confirmed in the present case, and the patient’s clinical features and overexpression of IGF2 allowed speculation regarding the specific subtype of molecular defect. This study suggests that characteristic clinical features and IGF2 staining may help to predict the subtype of molecular defects of 11p15.5 in patients with BWS.

## Data Availability

The data supporting the conclusions of this article are included within the article.

## Abbreviations

BWS: Beckwith–Wiedemann syndrome
IGF2: Insulin-like growth factor-2
pUPD: Paternal uniparental disomy
pUPD11: PUPD of chromosome 11
